# Bayesian model accounting for within-class biological variability in Serial Analysis of Gene Expression (SAGE)

**DOI:** 10.1186/1471-2105-5-119

**Published:** 2004-08-31

**Authors:** Ricardo ZN Vêncio, Helena Brentani, Diogo FC Patrão, Carlos AB Pereira

**Affiliations:** 1 Statistics Department, Instituto de Matemática e Estatística – Universidade de São Paulo, Rua do Matão 1010, 05508-090 São Paulo, BRAZIL; 2 BIOINFO-USP – Núcleo de Pesquisas em Bioinformática da Universidade de São Paulo, Rua do Matão 1010, 05508-090 São Paulo, BRAZIL; 3 Ludwig Institute for Cancer Research – São Paulo Branch, Rua Prof. Antônio Prudente 109, 01519-010 São Paulo, BRAZIL; 4 Hospital do Câncer A.C. Camargo, Rua Prof. Antônio Prudente 109, 01519-010 São Paulo, BRAZIL

## Abstract

**Background:**

An important challenge for transcript counting methods such as Serial Analysis of Gene Expression (SAGE), "Digital Northern" or Massively Parallel Signature Sequencing (MPSS), is to carry out statistical analyses that account for the within-class variability, i.e., variability due to the intrinsic biological differences among sampled individuals of the same class, and not only variability due to technical sampling error.

**Results:**

We introduce a Bayesian model that accounts for the within-class variability by means of mixture distribution. We show that the previously available approaches of aggregation in pools ("pseudo-libraries") and the Beta-Binomial model, are particular cases of the mixture model. We illustrate our method with a brain tumor vs. normal comparison using SAGE data from public databases. We show examples of tags regarded as differentially expressed with high significance if the within-class variability is ignored, but clearly not so significant if one accounts for it.

**Conclusion:**

Using available information about biological replicates, one can transform a list of candidate transcripts showing differential expression to a more reliable one. Our method is freely available, under GPL/GNU copyleft, through a user friendly web-based on-line tool or as R language scripts at supplemental web-site.

## Background

An important challenge in Serial Analysis of Gene Expression (SAGE) [1] analysis is the decision whether a gene is differentially expressed between two classes, for example tumoral vs. normal classes. In statistical terms, this essential step is to test the null hypothesis H_0_: "gene has no differential expression between the two probed classes". A much more usual approach is to assign an index (*P*-value or Bayes factor, for example) that measures the confidence/significance of the hypothesis and let the biologists themselves to establish a cutoff of what they call significant.

This necessity arises because counting sequenced SAGE tags is a process prone to random and systematic errors that affect gene expression abundance estimates. Systematic errors may come from various sources such as GC content bias [2], sequencing errors [3,4] as well as the possibility of non unique tags. This kind of error can be detected/corrected using some bioinformatics procedures such as quality control of automatic sequencing pipe-line [5], or statistical estimation procedures such as "denoising" [6,7]. Random errors are due to the inherent stochastic characteristic of SAGE data acquisition: sampling from automatic sequencing. Like colored balls in an urn, sampling and counting SAGE tags from a library is commonly modeled by a Bernoulli Process relying on an infinite population sampling approximation.

If an Expressed Sequence Tag (EST) library is non-normalized, its counting data, also known as "Digital-Northern", reflects the abundance of genes. Likewise, the Massively Parallel Signature Sequencing (MPSS) [8] technique counts tags to infer the transcriptome, but using a completely different strategy from traditional DNA sequencing methods, that allows augmented high-throughput capability. Therefore, all the results discussed here are readily applicable to "Digital-Northern" or MPSS context since, from a mathematical viewpoint, all represent the same bioinformatics problem: counting transcripts (as balls in urns).

Nowadays, the variability in SAGE abundance data is modeled only as due to sampling from sequencing, since almost all statistical procedures are performed after aggregation of observations from various libraries of the same class, creating a "pseudo-library". See [9-11] for good reviews on statistical techniques used in SAGE analysis. This extensively used trick tacitly ignores the within-class variability, i.e., the biological variability among individuals within a class (different patients having the same cancer diagnosis, for example), and could lead to overconfident conclusions.

## Results

Here we propose a Bayesian model of mixtures to account for within-class variability as a generalization of the Beta-Binomial model [12]. We also show that the usual "pseudo-library" construction is a particular case of our mixture model. Finally, we propose the use of the Bayes Error Rate to intuitively rank the differential expression hypothesis under a Bayesian framework, avoiding several technicalities and difficulties such as: typeI and typeII error analysis, Bonferroni-like multiple testing correction, asymptotic results evocation, imposition of a test statistic and null probability density function (pdf), and so on.

### Statistical model

The counting process from automatic sequencing of one single *i*-th library is often modeled as a Bernoulli Process and a fixed unknown tag abundance *π*_*i *_is implicitly assumed. The pdf of the random variable of interest, "expression abundance" *π *∈ [0;1] among all *n *libraries is unknown, thus each library could be regarded as being created by a realization of *π*. These features lead naturally to mixture models [13,14]:


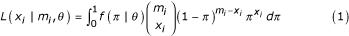


where: *f*(·) is the unknown pdf of the abundance among same-class libraries parameterized by a vector ***θ***, **X **= (*x*_1_,..., *x*_*n*_) is the vector of counts in all *n *libraries of same class, **M **= (*m*_1_,..., *m*_*n*_) is the vector of library sizes and *L *is the likelihood of each *i*-th observation.

The common procedure of merging all observations from libraries of the same class, constructing a "pseudo-library" before statistical inference, is recognized as a particular case of this mixture model: just assume that all libraries have strictly the same abundance, with no biological variability. Mathematically, this is a function with infinite probability density over one single abundance value *π *= *θ *and zero over every other *π *≠ *θ*, or a Dirac's Delta function. Using *f*(·) as a Dirac's Delta function constrained to [0;1], turn Eq.1 into the familiar and commonly used binomial distribution (see derivation in the Methods section).

We believe that Dirac's Delta is a naive description of real-life SAGE libraries. The Beta distribution is an alternative with non-zero within-class variance to account for intuitively expected biological differences among them. Using *f*(·) as a Beta in Eq.1, yields the so-called Beta-Binomial model (see derivation in the Methods section).

Given the parameter vector ***θ ***that describes the random variable *π *of some fixed gene *G*, we must decide if there is a difference between A and B classes (e.g, tumor vs. normal classes). We propose to consider genes as being differentially expressed based on non-superposition of the predictive Beta pdfs of both A and B classes. By "predictive" we mean that we use the *a posteriori *mode in the Beta pdfs. The "non-superposition" intuitive feature is mathematically written as the Bayes Error Rate *E *[15]:


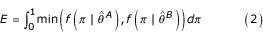


where *f*(·) is the Beta pdf and "hat" over the parameters indicate the values that lead to an *a posteriori *pdf maximum. The *a posteriori *distribution is obtained as usual from Bayesian Statistical Theory (*a priori *pdf choice and detailed derivation are in the Methods section).

**Figure 1:**
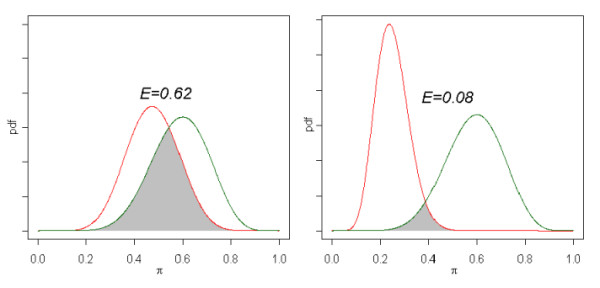
**The Bayes Error Rate illustration**. This figure shows two illustrations of the proposed use of Bayes Error Rate *E *to define differentially expressing genes based on pdf of expression abundance *π*. The left example shows an obvious superposition of classes' pdf, thus a gene having this profile does not present evidence of differential expression between classes. The right example shows two pdfs "far apart" and genes with this kind of behaviour should be safely considered differentially expressing between two classes.

Intuitively, if the pdfs are "far apart", the gene probably has reproducible differential expression between classes. In this case, rarely could one misclassify class A as B and vice-versa. Figure 1 gives some insight about this fact. Using our proposed approach, the "far apart" notion means a small Bayes Error Rate *E*. For adepts of the Frequentist Statistics, this evidence measure could resemble a typeI and typeII errors sum, however it is just an illustrative analogy.

As in any significance test method, the experimenter must define what is a high significance *E *value. This cutoff should be guided by external and independent confirmatory assays. To avoid crude decision boundaries, one could rank their significance results but there is no way to avoid some arbitrariness in any kind of statistical test.

In the classical Frequentist Statistics framework, it is common to call a result as significant if it presents a *P*-value ≤ 0.01 in a *t*-like-test, hoping that this could control the error at this level. However, due to technical difficulties such as lack of sensitivity of posterior confirmatory methods or high absolute expression (not differential expression) necessity, this apparent statistically sound results could be not useful in a pragmatic sense. That is why we prefer to rank the differential expression results and allow researchers to establish a cutoff compatible with their subsequent application for the selected genes, rather than split them based in assumption-derived error-rate cutoffs. People familiar with the Frequentist Statistics framework could miss multiple testing considerations, typeI/typeII error studies, and so on. However, in the Bayesian framework, several of these concerns are meaningless since we work with parameters space and not with sample space. The bayesians avoid statements about "data that could be observed but was not" and work only with available information (prior and experimental), extracting all possible information from data effectively observed.

For those genes classified as differentially expressed, one should aggregate intuitive information adding "error-bars" to expression ratios. Recently we have developed a method to add credibility intervals to gene expression ratio [16], which could improve posterior analyses such as clustering [17] or comparison with microarray data.

### Comparison with available methods using publically available data

To show the model is usefulness, we applied it to a tumor vs. normal two-classes comparison problem. We chose a subset of brain tumor SAGE data from The Cancer Genome Anatomy Project's SAGE Genie public database web-site [18]. The SAGE Genie performs several bioinformatics protocols to assure the quality of its data with systematic errors cleaning/correction [19].

We used all 4 available libraries in SAGE Genie until Jan/2004 from astrocytoma grade III tumors and almost all (except the fetal library) normal brain libraries (see Methods section for details about libraries).

We want to stress 3 typical and important cases: (i) when our measure agreed with other evidence measures accepting null hypothesis H_0_, i.e., there is no evidence of differential behavior between tumor and normal classes; (ii) when our method agreed with others rejecting H_0_, i.e., there is evidence of differential expression; and (iii) when our method showed evidence in favor but other evidence measures showed evidence against the H_0_. Case (iii) is the main motivation of our method since it reveals situations that researchers may call a gene differentially expressed and, in fact, it could be not so significant if biological replicates are taken into account. The other evidence measures used were: the Audic-Claverie bayesian evidence [20], the classical Fisher Exact Test *P*-value, and the classical *χ*^2 ^*P*-value, all obtained using the IDEG6 web-interface [21,22] (see Methods section).

**Figure 2:**
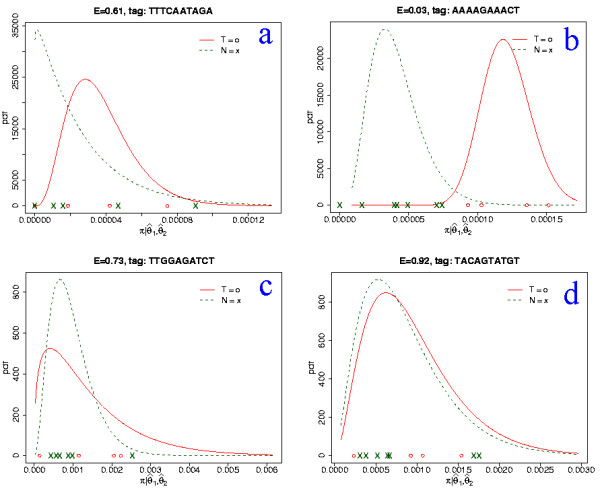
**Maximum *a posteriori *Beta pdfs of classes in main prototype cases**. The predictive pdf and the Bayes Error Rate *E *of both tumoral (T) and normal (N) classes, for examples tags contained in the three important prototype cases described in text, are shown in the figure. The 'x' and 'o' marks represent observed abundances in each tumoral and normal. Frame a) shows case (i) example when methods agree with "no differential expression" conclusion. Frame b) shows case (ii) example when methods agree with "differential expression" conclusion. Frame c) and d) show case (iii) examples when classical *P*-value method leads to significant differential expression between classes and our method indicates pdf superposition if one take within-class variability into account. Individual observations indicate that the classes are not clearly divided, casting doubt on "differential expression" conclusion.

A case (i) prototype is the TTTCAATAGA tag with **X**_T _= (0, 2, 5, 8) and **X**_N _= (1, 1, 0, 0, 0, 7, 2). The Audic-Claverie, Fisher and *χ*^2 ^methods yield *P*-values of 0.06, 0.44, 0.41, respectively, indicating low evidence against H_0 _for all mystical significance level cutoffs ≤ 0.01, ≤ 0.05 or ≤ 0.1. The Bayes Error Rate evidence is *E *= 0.61, an intuitively unacceptable superposition level between the normal and tumoral predictive Beta pdfs, showing that there is no separable behavior between classes. Figure 2a shows an obvious superposition between pdf and observations of this two classes.

A case (ii) prototype is the AAAAGAAACT tag with **X**_T _= (7, 11, 18, 10) and **X**_N _= (7, 1, 2, 1, 2, 0, 3). All *P*-values are 0.00 (zero), significant at any cutoff level. Our evidence is *E *= 0.03, showing safely that this gene behaves differentially between normal brain and astrocytoma grade III patients. Figure 2b shows that two Betas are apart from each other and, even observing clear within-class variability, the expression is different.

A case (iii) prototype is the TTGGAGATCT tag with **X**_T _= (7, 239, 244, 123) and **X**_N _= (54, 27, 33, 21, 40, 196, 28). All *P*-values are 0.00 (zero), indicating significant difference between classes. On the other hand, our evidence *E *= 0.73 indicates high superposition between tumor and normal classes. Figure 2c shows that within-class variability for tumor class is not negligible. It is obvious that individual libraries confound their results with normal brain libraries, and the Betas have a relatively high intersection. Using a common "pseudo-library" approach, one is lead to call this gene as a strong discriminator between classes. We believe that this is a suspect conclusion.

There are several other obvious case (iii) examples, such as tag TACAGTATGT in Figure 2d, that received *P*-values < 0.01 from all other methods, and they are the main concern of our method since they may lead to waste of resources in clinical validation efforts of genes that, by SAGE itself, could be left behind in favor of other promising genes. All tag results are available as additional file and graphics for all tags are at the supplemental web-site [23].

One could think about a case (iv) when considering within-class variability leads one to H_0 _rejection, but considering "pseudo-libraries" leads to H_0 _acceptance. This seems to be inconsistent since one expects that, once H_0 _is accepted in a simplified model, it should also be accepted in the complete model. In fact, we do not observe such a situation, except by tags with *P*-values or Bayes Error Rate very close to arbitrarily defined cutoff values. We believe that these occurrences are just "edge effect" manifestations.

## Discussion

In order to assure that we are dealing with a fundamental question in SAGE analysis, we show more insights analyzing the method's robustness using the same data but excluding "small" libraries. Also, we draw some parallels between our proposed method and the only available published solution for dealing with within-class variability, a *t*-test approximation [12].

**Figure 3:**
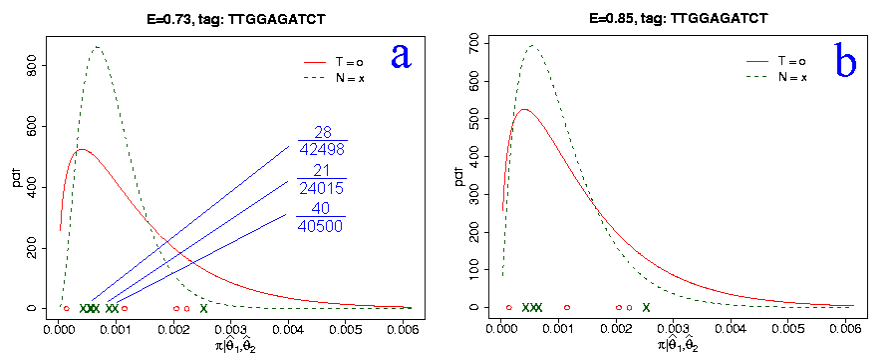
**Effect of "small" size libraries on the final result. **The predictive pdf and the Bayes Error Rate *E *of both tumoral (T) and normal (N) classes for examples tags contained in the three important prototype cases described in text are shown. The 'x' and 'o' marks represent observed abundances in each tumoral and normal libraries. Frame a) shows the result using all libraries. The "small" libraries (size < 50,000) are highlighted with their counts over library size. Frame b) shows results excluding those "small" libraries. It is clear that results are pretty much the same and that "small" libraries are not (necessarily) outliers of the sampling.

We used our method with all available libraries but some of them are smaller than 50,000 tags (see Table 1). In the SAGE community, libraries smaller than this arbitrary limit are considered "small". Several researchers claim that these are non-representative and should be excluded from analysis. We observed several case (iii) tag examples which remain as case (iii) if we use libraries with size > 40,000 and > 50,000 (shown at the supplemental web-site only). Figure 3 shows a tag example analyzed in these tree setups and it is clear that inclusion of "small" libraries gave pretty much the same result, indicating robustness of our method against small class size variations and against "small" sized libraries. Moreover, these libraries are not always outliers from biological sampling but seem to be samples like any other. These results suggest that one can use the "small" libraries, jointly with non-"small" ones, because biological variability seems to be greater than binomial sampling variability.

Obviously, we are not recommending to use only "small" libraries in SAGE analysis, but suggesting that our method is relatively robust. For low expression genes, the binomial sampling variability should become more relevant as the library size decreases. Also, the results obtained using two/three libraries could be very different from using just one. These proprieties could be tag dependent since some tags could be much noisier than others for biological reasons. Some "denoising" procedure could be used before application of our method [7]. Therefore, our findings should be carefully interpreted.

To prove that the incoherence of using "pseudo-libraries" methods is not a prerogative of tags showing small fold-changes, we analyzed another three very illustrative examples: ATGGCAACAG, GGATGTGAAA, and GTATGGGCCC; which are case (iii) tags. These tags present high fold changes: 7.59, 8.15 and 25.80 fold-change respectively, augmented in pooled tumor libraries. Using the well-known Fisher Exact test, *χ*^2 ^classical test and the Audic-Claverie's method, we get 0.00 (zero!) for all *P*-values of the no differential expression null hypothesis. Using the conceptually different Bayesian *P*-value implemented at SAGE Genie [24,25] we obtain 0.01, 0.00 and 0.00 respectively for posterior probabilities of fold-changes greater than 4-fold. Finally, using our own proposed measure, applied to the pool, we get *E *= 0.00 meaning no superposition between the two classes pdfs. All these results indicate strong significance in differential expression of these tags.

However, if we consider within-class variability, the test proposed by Baggerly *et al. *[12] yields 0.08, 0.07 and 0.15 respectively for *t*-test *P*-values, and our method yields Bayes Error Rates of 0.38, 0.37, 0.43 respectively; indicating not so significant evidence in favor of the differentially expressed hypothesis. A closer look at the graphics of these tags induces one to believe that there is no reproducible differential expression because several observations of tumor and normal are superimposed (all graphics available at supplemental web-site [23]).

**Figure 4:**
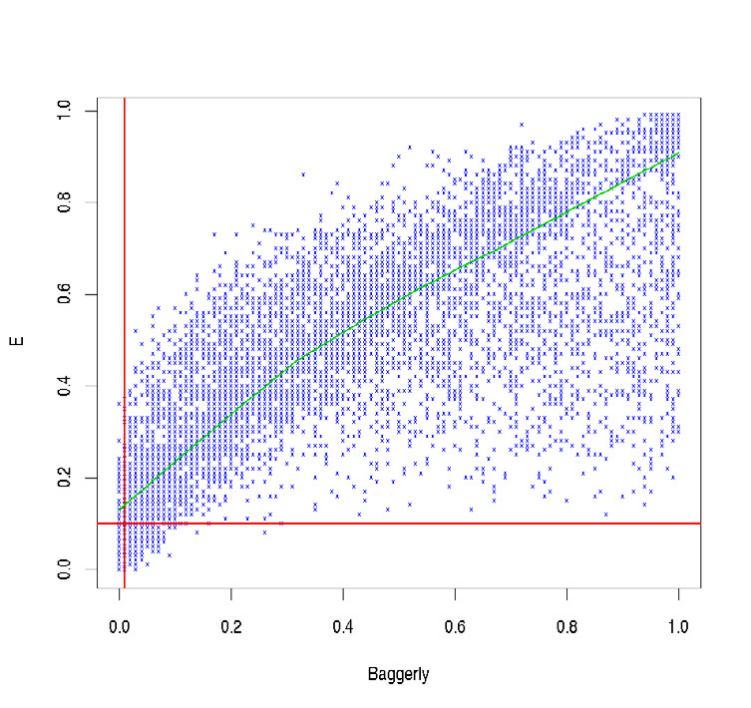
**Qualitative comparison of Bayes Error Rate and *t*-test approximation. **It is shown the Bayes Error Rate (*E*) versus the *t*-test approximation *P*-value (Baggerly) [12] for each tag. The red lines are arbitrary cutoffs that define significance regions E ≤ 0.1 and Baggerly ≤ 0.01. The green line is a LOWESS trend fit.

Since we show clearly that methods that use "pseudo-library" aggregation could be incoherent in some cases, a natural question is how our proposed method performs compared to the only published solution that accounts for within-class variability, the Baggerly *et al. *[12]*t*-test approximation. Without knowing the true state of all tags, it is impossible to carry out a serious benchmark. Since the interpretation of evidence measures is very different, the performance could be subjected to an arbitrary cutoff selection for each method. Figure 4 shows a scatter-plot of evidence measures obtained for each of the two methods.

It is clear from this graphic that there are many more tags considered as differentially expressed using our method than the *t*-test approximation, considering *E *≤ 0.1 and *P*-value ≤ 0.01. There are also some tags selected by *t*-test and ignored by ours. It is impossible to know which method perform better without the true unknown status of those tags. Looking at individual libraries results, constructed as depicted in Figure 2 for example, could help in this analysis but this is a subjective procedure.

It is important to bear in mind that a difficulty is hidden in the Beta modeling imposed in the very first beginning. If Beta is not a good model for an unknown biological behavior, then some apparent inconsistency could appear in both Baggerly *et al. *[12] and our approaches. However, our general mixture model allows another propositions. Other simplex constrained pdfs, different from Beta, exist but the tractability is much more difficult [26]. We believe that to build a fully non-parametric approach to this problem is a very hard issue, but should be considered as a future challenge.

## Conclusion

Until now, almost all statistical methods for SAGE data analysis tacitly ignore the within-class variability. To our knowledge, the firsts to formally address this issue was Baggerly *et al. *[12] who introduced the Beta-Binomial model as the correct way to model the probability of counting tags instead of Binomial models. They also proposed a *t*-like statistics, outlined a possible hypothesis test using the classical Frequentist Statistics framework and evoked some asymptotic results for *t *pdf justification.

In this work we presented the Bayesian alternative for this problem and defined a theoretical model that views Baggerly's Beta-Binomial approach or even the common Binomial approach as particular cases of mixture models. Other models are possible modifying the mixing distribution, such as Beta-Poisson [14], or using other simplex constrained pdf [26] to model expression abundance. At last, but not at least, we proposed a method for ranking differentially expressed genes between two classes using the Bayes Error Rate as an intuitive measure of separation between the classes pdfs, avoiding statistical test formalism and its conceptual/practical difficulties.

We show that there are cases in which approaches that ignore within-class variability will lead to high significance in differences between tumor and normal classes, but looking carefully at individual observations jointly, one should not attribute such high significance to them since abundance probability density functions have considerable superposition.

In conclusion, we recommend that within-class variability must be taken into account in any statistical analysis of SAGE data if replicates are available. We suggest that biological replication should be considered in planning new SAGE experiments.

## Methods

### General bayesian model

To start generically, suppose that the probability density function (pdf) for the random variable of interest "expression abundance" *π *∈ [0;1] of some gene *G *is indexed in a model family by means of a parameter vector ***θ***. Therefore, following the usual Bayesian framework, the *a posteriori *pdf that describes the class is:


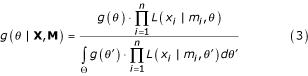


where: **X **= (*x*_1_,..., *x*_*n*_) is the vector of counts in all *n *libraries of same class, **M **= (*m*_1_,..., *m*_*n*_) is the vector of total observations in all *n *libraries of same class, *g*(·) is the *a priori *pdf, and *L *is the likelihood of each *i*-th observation. Note that the product of all likelihood functions over all observations is the so-called Likelihood Function.

The counting process from automatic sequencing is often modeled as a Binomial. Since the sample size and the stopping rule are not known in advance the model is not strictly Binomial. We do not need the combinatorial constant in the model, but we write it just because it is commonly used and will vanish in *posteriori *expression anyway.

### "Pseudo-Library" method as particular case

Merging all observations from the same class libraries and constructing "pseudo-libraries", with the sum of their components, is the standard procedure to use replicates. Our general model is reduced to this (unrealistic) one if one uses *f*(·) as a Dirac's Delta in Eq.1:


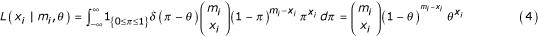


where: **1**{·} is the indicator function.

Using Eq.4 in Eq.3 yield:


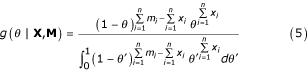


where: g(*θ*) = 1, the non-informative uniform *a priori *distribution.

The expert recognizes that *θ *~ Beta(1 + **Σ***x*_*i*_, 1 + **Σ***m*_*i *_- **Σ***x*_*i*_), and the sum of observations is the mathematical translation of "pseudo-libraries" construction.

### Beta-Binomial method as particular case

The only published solution that allows non-zero within-class variance in SAGE analysis is the Beta-Binomial model [12]. Using *f*(·) as a Beta in Eq.1 we get the Beta-Binomial model as a particular case of general model:


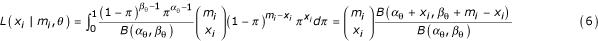


where: *B*(·) is the beta special function, and:


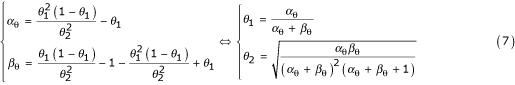


Again, an expert recognizes ***θ*** = (*θ*_1_, *θ*_2_) as the mean and standard deviation (stdv) of a Beta random variable. We prefer this parameterization of Beta distributions instead of the common (*α*, *β*) one because: (i) it is much more intuitive to biologists to deal with mean and stdv than with abstract *α *and *β*, and (ii) as *α*, *β *> 0, the domain Θ = {(*θ*_1_, *θ*_2_): 0 ≤ *θ*_1 _≤ 1, 0 ≤ *θ*_2_^2 ^<*θ*_1 _(1- *θ*_1_) ≤ 1/4} is bounded and much more amenable to perform the necessary numerical computations.

Using Eq.6 in Eq.3 yield:


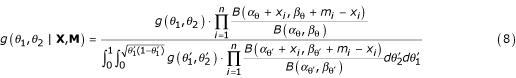


where: *g*(*θ*_1_, *θ*_2_) is the *priori *pdf.

### *A Priori *distribution definition

To complete a Bayesian model, it is necessary to choose the *a priori *pdf. We use an uniform distribution over Θ. On the other hand, we know in advance that variance of this model cannot be smaller than the variance eventually obtained if we do not consider within-class variability. Even if the within-class variability is very small, it cannot be estimated as being smaller than the simple sampling error because they are inseparable, and sampling error is the lower limit [12]. As an illustration, the same situation could occur if one takes several diameter measurements of a folded paper ball and a perfect sphere using a common ruler. In the first case, the intrinsic nature of the measured object dominates the measurement variability but, in the second case, we cannot know the diameter of the perfect sphere with better precision than our ruler can measure.

This kind of knowledge is naturally incorporated in Bayesian statistics by means of *a priori *distributions. To match our desired features, it is sufficient to define an uniform over the Θ parameter space but constrained at a minimum stdv σ, obtained from the result of no within-class variance model:


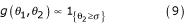


over domain Θ = {(*θ*_1_, *θ*_2_): 0 ≤ *θ*_1 _≤ 1, 0 ≤ *θ*_2_^2 ^<*θ*_1 _(1 - *θ*_1_) ≤ 1/4}.

Since we showed (Eq.5) that the no within-class variance model is *θ *~ Beta(1 + Σ*x*_*i*_, 1 + Σ*m*_*i *_- Σ*x*_*i*_), it is easy to obtain our lower stdv boundary from Beta variance:


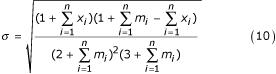


Therefore, using Eq.9 and Eq.10 in Eq.8, our *posteriori *is completely defined.

### Differential expression detection

We detect a tag as differentially expressed using the Bayes Error Rate *E *[15] in both predictive Beta pdfs:


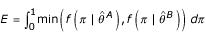


where:


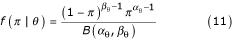


Note that *f*(·) is the Beta pdf, as in Eq.6 development. The "hat" over ***θ*** = (*θ*_1_, *θ*_2_) indicates values that leads Eq.8 to maximum. As usual, the maximization, subject to constrain Θ defined previously, is made upon logarithm of *posteriori's *core since it gives the same estimates as the *posteriori *itself:


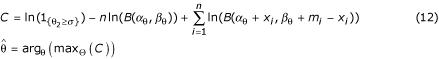


**Figure 5:**
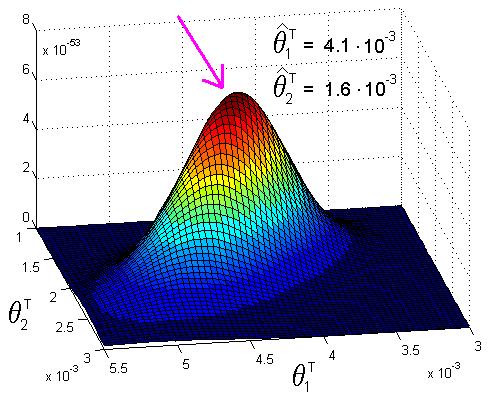
**Illustration of Maximum *a Posteriori *parameter estimation. **The maximum a posteriori parameters of Eq.12 in an artificial example it is shown. The bi-dimensional pdf from which "hat" (pointed by the arrow) parameters are extracted is proportional to that described in Eq.8.

Figure 5 shows an example of this process. See Results section to get an intuitive notion of this evidence measure.

### Implementation – numerical analysis

The method was implemented as R language [27,28] scripts which are freely available under GPL/GNU copyleft license at supplemental web site [23]. At this web page there are details on how to run it locally.

Our method is computer-intensive mainly because some numeric maximization and integration are needed. We used efficient R built-in routines to perform such numerical tasks. Remember that maximization needed in Eq.12 is constrained, thus we used simply auxiliary re-parameterization to obtain linear constrains and used the constrOptim R routine. For numerical integration we used the 1-dimensional gaussian quadrature integrate R built-in function. Although numerical integration of Eq.9 should be performed in all [0;1] support, the relevant contribution for this integral is concentrated in a much smaller region. Integrating over the formal limits will cause serious numerical errors, and to avoid this problem we approximate our integration region to an interval delimited by 0.005 and 0.995 quantile of each Beta pdf since the relevant density lie in there.

The credibility intervals ("error-bars") for the expression ratio of interesting tags were obtained as described in our recent work [16]. We chose arbitrarily 68% credibility intervals.

### Implementation – Web based interface

**Figure 6:**
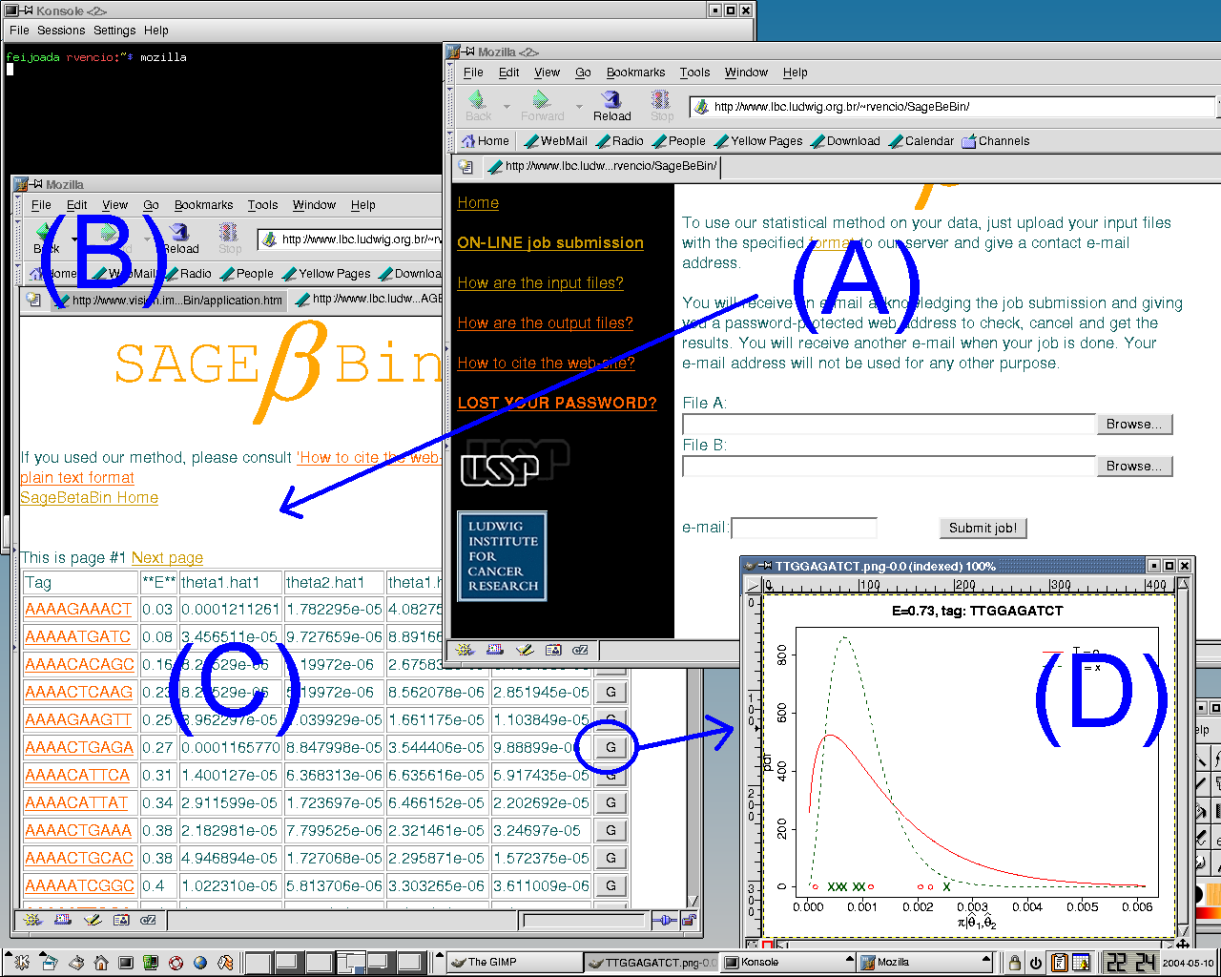
**Snapshot of the web-interface for our SAGEbetaBin method. **An illustration of on-line tool implemented to make our method easily available it is shown. Researchers submit their data (A) and receive, by e-mail, an alert when we finished the job along with instructions to get results in a password-protected web-page (B). If the ID supplied is a human tag (C), then results are linked with SAGE genies' tag-to-gene map. The individual observations graphics, as utilized in this work, is available on-demand (D).

We have also developed an easy-to-use web-based service that performs all calculations at our server and provides password-protected results. Although desirable, for the sake of automatic web hyperlink with SAGE Genie database, it is not necessary to explicitly identify the tags analyzed but rather any (custom) i.d. string. This could increase privacy or make our web-interface useful for "Digital-Northern", MPSS or any mathematically related problem of mixtures from binomial sampling. Figure 6 shows snapshots of the interface.

### Publically available data

The Table 1 list the SAGE Genie's library name, Gene Expression Omnibus (GEO) [29] accession code and size of all used libraries.

For our aims, it is sufficient to focus the analysis at the tag level. Thus, we process the tag counts and let the identification of tag's best gene match as a posterior question that could be carefully done only to really interesting tags. We choose not to process tags whose counts appear only in libraries of one class. It is important to note that all libraries are from bulk material, without cell-lines, and came from patients with similar disease description. The normal libraries came from different normal regions of the brain.

We think that this data set is very illustrative since there are biological replicates in the tumor class allowing clear verification of within-class biological variability. On the other hand, taking only one kind of disease, astrocytoma grade III, instead of all brain tumors in the database, leads one to believe that the within-class variability is in fact due to biological diversity of the patients and not due to very distinct molecular profile of distinct brain tumors stored in SAGE Genie's database.

Therefore, we believe that this *in silico *comparison is well-suited to demonstrate the necessity of dealing with within-class effect, although it is not our aim here to make a detailed or biological analysis of brain tumor data.

### Comparison with other methods

To bring some intuition about our differential expression evidence measure, we tabulated evidence measure obtained from the famous Fisher Exact Test, the classical Pearson's *χ*^2 ^proportion test and the bayesian Audic-Claverie's method. All these tests were performed using the easy-to-use web-interface IDEG6 [21,22].

The "*P*-values" are conceptually very different from our evidence measure but are the most used evidence measures. Although numbers cannot be compared, the conclusions obtained from these methods should be since graphical representation of each library observation gives clear indication of incoherence of "pseudo-library" methods. The results of the significance measures for all tags are available as additional Excel^© ^or OpenOffice^© ^interactive files in which the user can set cutoffs for the significance measures, and explore the differences in conclusions.

We carry out a qualitative comparison of our method with Baggerly *et al. *[12]*t*-test approximation in a graphical way since it is impossible to judge them without the unknown true status of analyzed tags, given the too different interpretation of numeric values returned. In their Frequentist framework, the estimator *p*_*i *_= *x*_*i*_/*m*_*i *_is used for *π*_*i *_and a linear combination of these abundances is proposed as the correct way to combine results from different libraries:


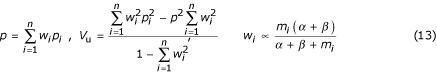


where *w*_*i *_are the weights that yield an unbiased minimum variance estimator *V*_*u *_for weighted proportion's variance and ***θ*** = (*α*, *β*) are the Beta pdf parameters. However, this unbiased variance could be unrealistically small when it becomes smaller than the sampling variability. We know that the variance of this model cannot be smaller than the variance eventually obtained if we do not consider within-class variability. Therefore, they propose the final *ad hoc *estimator:

*V *= max [*V*_*u*_; *V*_*pseudo-lib*_]     (14)

where:


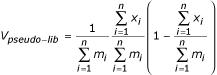


The max(·) function assure that *V *is not unrealistic small when *V*_*u *_is unrealistic small. To *fit *all these parameters, they used the computationally practical Method of Moments. Once *p*_A_, *p*_B_, *V*_A _and *V*_B _are found for classes A and B, these authors test if the proportions are significantly different proposing the use of a *t*_*w *_statistics as following a Student's *t*_*df *_pdf:


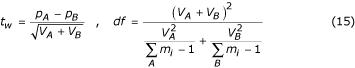


## List of Abbreviations

SAGE: Serial Analysis of Gene Expression

MPSS: Massively Parallel Signature Sequencing

EST: Expressed Sequence Tag

pdf: probability density function

GEO: Gene Expression Omnibus

## Authors' Contributions

RV conceived and executed this work. HB helped with all biological issues. DFCP helped in differential expression detection methods and implemented the on-line web-based tool. CABP helped with Bayesian statistics and proposed the mixture ideas.

## Supplementary Material

Additional File 1 **Results for all evidence measures**. This file allows the user to interactively define significance cutoffs for ranked tags. The ranks are based on evidence measures against "no differential expression" hypothesis, i.e., evidences closer to 0 (zero) denote higher confidence in differential expression and closer to 1 (one) denote no evidence of differential expression. Click here for file

## Figures and Tables

**Table 1:** Brain tumor and normal libraries from SAGE Genie used as real data application.

**#**	**Library Name**	**GEO accession**	**Total Tags**
1	SAGE_Brain_astrocytoma_grade_III_B_H1020	GSM697	51573
2	SAGE_Brain_astrocytoma_grade_III_B_H970	GSM14763	106982
3	SAGE_Brain_astrocytoma_grade_III_B_R140	GSM14773	118733
4	SAGE_Brain_astrocytoma_grade_III_B_R927	GSM14766	107344
5	SAGE_Brain_normal_cerebellum_B_1	GSM761	50385
6	SAGE_Brain_normal_cerebellum_B_BB542	GSM695	40500
7	SAGE_Brain_normal_cortex_B_BB542	GSM676	94233
8	SAGE_Brain_normal_cortex_B_pool6	GSM763	62451
9	SAGE_Brain_normal_peds_cortex_B_H1571	GSM786	77554
10	SAGE_Brain_normal_substantia_nigra_B_1	GSM14796	42498
11	SAGE_Brain_normal_thalamus_B_1	GSM713	24015
